# MAPK signaling pathways and HDAC3 activity are disrupted during differentiation of emerin-null myogenic progenitor cells

**DOI:** 10.1242/dmm.028787

**Published:** 2017-04-01

**Authors:** Carol M. Collins, Joseph A. Ellis, James M. Holaska

**Affiliations:** University of the Sciences, Department of Pharmaceutical Sciences, 600 S. 43rd St, Philadelphia, PA 19104, USA

**Keywords:** Cell signaling, Emerin, Emery-Dreifuss muscular dystrophy, Lamin, Myogenic differentiation

## Abstract

Mutations in the gene encoding emerin cause Emery–Dreifuss muscular dystrophy (EDMD). Emerin is an integral inner nuclear membrane protein and a component of the nuclear lamina. EDMD is characterized by skeletal muscle wasting, cardiac conduction defects and tendon contractures. The failure to regenerate skeletal muscle is predicted to contribute to the skeletal muscle pathology of EDMD. We hypothesize that muscle regeneration defects are caused by impaired muscle stem cell differentiation. Myogenic progenitors derived from emerin-null mice were used to confirm their impaired differentiation and analyze selected myogenic molecular pathways. Emerin-null progenitors were delayed in their cell cycle exit, had decreased myosin heavy chain (MyHC) expression and formed fewer myotubes. Emerin binds to and activates histone deacetylase 3 (HDAC3). Here, we show that theophylline, an HDAC3-specific activator, improved myotube formation in emerin-null cells. Addition of the HDAC3-specific inhibitor RGFP966 blocked myotube formation and MyHC expression in wild-type and emerin-null myogenic progenitors, but did not affect cell cycle exit. Downregulation of emerin was previously shown to affect the p38 MAPK and ERK/MAPK pathways in C2C12 myoblast differentiation. Using a pure population of myogenic progenitors completely lacking emerin expression, we show that these pathways are also disrupted. ERK inhibition improved MyHC expression in emerin-null cells, but failed to rescue myotube formation or cell cycle exit. Inhibition of p38 MAPK prevented differentiation in both wild-type and emerin-null progenitors. These results show that each of these molecular pathways specifically regulates a particular stage of myogenic differentiation in an emerin-dependent manner. Thus, pharmacological targeting of multiple pathways acting at specific differentiation stages may be a better therapeutic approach in the future to rescue muscle regeneration *in vivo*.

## Abstract (precis)

**Editors' choice:** HDAC3, p38 MAPK and ERK signaling are altered during differentiation of myogenic progenitors lacking emerin; pharmacological activation or inhibition of these signaling proteins rescues specific stages of myogenic differentiation.

## INTRODUCTION

The nuclear envelope is composed of two lipid bilayers and functionally separates the nucleoplasm from the cytoplasm. Embedded within the nuclear envelope are the nuclear pore complexes, which provide bi-directional transport across the nuclear membrane. The outer nuclear membrane of the nuclear envelope is contiguous with the endoplasmic reticulum. The outer nuclear membrane bends around the nuclear pore complex at its insertion site to form the inner nuclear membrane (Dittmer and Misteli, 2011; Simon and Wilson, 2011).

Although the outer and inner nuclear membranes arise from a common membrane, they are functionally distinct membranes containing proteins localizing specifically to either the outer or inner nuclear membrane. The inner nuclear membrane of the nuclear envelope contains a large number of integral inner nuclear membrane proteins (Gruenbaum and Foisner, 2015). There are more than 130 inner nuclear membrane proteins, although specific cell types express only a subset of these inner nuclear membrane proteins (de Las Heras et al., 2013; Gonzalez et al., 2012; Korfali et al., 2010, 2012; Malik et al., 2010; Schirmer et al., 2003; Wilkie et al., 2011; Worman and Schirmer, 2015). These inner nuclear membrane proteins have diverse cellular roles, including maintenance of nuclear structure, genomic organization, chromatin architecture, regulating gene expression, cell cycle regulation and cytoskeletal organization (Barton et al., 2015; Holaska, 2016). Underlying the inner nuclear membrane is a network of Type V intermediate filament proteins named lamins that provide nuclear rigidity and elasticity (Burke and Stewart, 2013; Dahl and Kalinowski, 2011; Ho and Lammerding, 2012). The nuclear lamins are also required for the localization of integral inner nuclear membrane proteins. The nuclear lamins and its associated inner nuclear membrane proteins define the nuclear lamina.

Emerin is a ubiquitously expressed integral inner nuclear membrane protein (Manilal et al., 1996; Nagano et al., 1996; Tunnah et al., 2005) with diverse roles in nuclear structure, chromatin architecture, genomic organization, cell signaling, and gene expression (Dedeic et al., 2011; Demmerle et al., 2012, 2013; Haraguchi et al., 2004; Holaska et al., 2004, 2006; Holaska and Wilson, 2006, 2007; Koch and Holaska, 2012; Markiewicz et al., 2006). Mutations in the gene encoding emerin cause X-linked Emery–Dreifuss muscular dystrophy (EDMD1), an inherited disorder causing progressive skeletal muscle wasting, irregular heart rhythms and contractures of major tendons (Bione et al., 1994; Méndez-López and Worman, 2012; Vlcek and Foisner, 2007; Worman, 2012). The autosomal dominant form of EDMD, EDMD2, which is caused primarily by mutations in *LMNA*, has similar phenotypes. Impaired skeletal muscle regeneration caused by the inability of skeletal muscle stem cells to differentiate is predicted to contribute to the skeletal muscle defects in EDMD.

Muscle regeneration is a multi-step process that repairs damaged muscle (Segales et al., 2016). Upon muscle injury, myogenic progenitor cells are activated and begin proliferating. A fraction of these cells will maintain their gene expression program and replenish the progenitor cell niche (Chang and Rudnicki, 2014). The remaining activated progenitor cells differentiate to become proliferating myoblasts. The proliferating myoblasts will then differentiate and form myocytes that move to the site of injury, fuse to the myofiber and repair the damaged muscle. Coordinated temporal expression of critical differentiation factors (e.g. MyoD, Pax3, Pax7, Myf5, myogenin) is required for proper differentiation and muscle regeneration (Chang and Rudnicki, 2014; Segales et al., 2016).

Multiple lines of evidence implicate impaired skeletal muscle regeneration in the skeletal muscle wasting seen in EDMD. Unlike Duchenne or Becker muscular dystrophies, increased skeletal muscle necrosis is rarely seen in EDMD patients, including increased skeletal muscle fiber permeability (Bonne et al., 2015). Severely affected EDMD patients exhibit extensive fibrosis due to the inability to regenerate and repair the damaged muscle. Emerin-null mice exhibit delayed skeletal muscle regeneration and repair, motor coordination defects, and mild atrioventricular conduction defects (Melcon et al., 2006; Ozawa et al., 2006). Emerin-null primary mouse myoblasts and emerin-downregulated myoblasts have impaired differentiation into multinucleated myotubes (Frock et al., 2006; Huber et al., 2009). Skeletal muscle biopsies from EDMD patients and emerin-null mice showed increased expression of genes important for skeletal muscle regeneration (Bakay et al., 2006; Melcon et al., 2006). The coordinated temporal expression of crucial differentiation genes, including *Myod1*, *Myf5*, *Pax3* and *Pax7*, is disrupted in emerin-null myogenic progenitors (Demmerle et al., 2013). Disruption of the coordinated temporal expression of these genes is caused by the failure of these genomic loci to properly localize to the nuclear periphery upon repression during differentiation. (Bakay et al., 2006; Koch and Holaska, 2012; Melcon et al., 2006).

Growing evidence suggests that emerin regulates signaling pathways important for myogenic differentiation. The Wnt, IGF-1, TGF-β and Notch signaling pathways, which are all important molecular pathways regulating myogenic differentiation and muscle regeneration, are disrupted in emerin-null myogenic progenitors (Conboy and Rando, 2002; Edwall et al., 1989; Jennische and Hansson, 1987; Jennische et al., 1987; Koch and Holaska, 2012; Massague et al., 1986; Polesskaya et al., 2003; Ridgeway et al., 2000). These molecular pathways have well-defined roles in maintaining satellite cell quiescence, satellite cell activation and myogenic differentiation during injury (Brack et al., 2008; Chang and Rudnicki, 2014; Rosenthal and Cheng, 1995; Segales et al., 2016). JNK, MAPK, ERK and NF-κB signaling pathways are also disrupted in emerin-downregulated myoblasts (Muchir et al., 2007a,b). The ERK pathway is upregulated in emerin-null cells and lamin-A R453W mutant cells (Favreau et al., 2008; Koch and Holaska, 2012; Muchir et al., 2007a,b). C2C12 myoblasts downregulated for emerin also had impaired differentiation, which was partially rescued by treatment with the ERK inhibitor U0126 (Huber et al., 2009). C2C12 myoblasts expressing the EDMD2-causing R453W *LMNA* mutation differentiate poorly and another ERK inhibitor, PD98059, partially rescued the impaired myogenic differentiation (Favreau et al., 2008). Inhibition of ERK signaling also prevented dilated cardiomyopathy in both EDMD1 and EDMD2 mouse models (Muchir et al., 2007a, 2012, 2014, 2009b; Muchir and Worman, 2016; Wu et al., 2014).

Proper temporal regulation of p38 MAPK signaling is also crucial for myogenic differentiation (Mozzetta et al., 2011; Palacios et al., 2010; Wu et al., 2000). RNA expression profiling of emerin-null myogenic progenitors revealed that the p38 MAPK pathway is activated in emerin-null myogenic progenitors (Koch and Holaska, 2012), suggesting that inhibition of p38 MAPK may rescue myogenic differentiation of emerin-null cells.

These previous studies support a model whereby disruption of these myogenic signaling pathways in emerin-null and emerin or lamin mutant myoblasts is responsible for their impaired differentiation. Here we use, for the first time, a pure population of emerin-null myogenic progenitors to test this hypothesis. These cells have many advantages over C2C12 myoblasts. C2C12 myoblasts used in most labs are more differentiated than myogenic progenitors, since they often aberrantly express lamin A, which should not be expressed in undifferentiated cells (Burattini et al., 2004; Hieter and Griffiths, 1999; Lattanzi et al., 2003; Leitch, 2000; Muchir et al., 2009b). Thus C2C12 differentiation may not be the best system for studying the early stages of myogenic differentiation. C2C12 myoblasts also exhibit aneuploidy and polyploidy for many genomic loci, including myogenic loci (Burattini et al., 2004; Easwaran et al., 2004; Leitch, 2000), because decades in cell culture have caused C2C12 myoblasts to diverge significantly from the myoblasts they were derived from. This polyploidy has the potential to generate artifacts and flawed data. Thus, any conclusions generated using C2C12 myoblasts to study cell signaling and chromatin regulatory mechanisms for myogenic differentiation may be inaccurate.

Another advantage of our cell system is that the emerin-null myogenic progenitor cells used in this study lacked emerin expression throughout development. Previous experiments analyzing the role of emerin in myogenic differentiation studied the effects of acute knockdown of emerin in C2C12 myoblasts, thereby creating additional potential artifacts caused by the continued low-level expression of emerin during differentiation. Emerin-null myogenic progenitors used in this study more accurately reproduce the chronic loss of emerin that occurs in EDMD1 patients, since patients lack emerin throughout development.

## RESULTS

### Emerin-null myogenic progenitors have impaired differentiation

Emerin-null myogenic progenitors were plated at high density and differentiation was induced by serum withdrawal. Three assays were used to monitor myogenic differentiation: cell cycle exit, myosin heavy chain (MyHC) expression and cell fusion into myotubes. Incorporation of EdU into the DNA of cycling cells was used to determine the percentage of cells in the cell cycle, while immunofluorescence microscopy with an antibody against MyHC determined the number of cells expressing MyHC. The differentiation index was defined as the percentage of cells containing three or more nuclei and expressing MyHC.

**Fig. 1.**
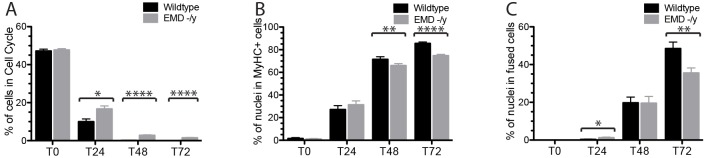
**Emerin-null myogenic progenitors exhibit impaired differentiation.** Wild-type (black) or emerin-null (EMD -/y, gray) myogenic progenitors were induced to differentiate by serum withdrawal and differentiation was assessed every 24 h. (A) Cell cycle withdrawal was monitored by measuring the incorporation of EdU. (B) Myosin heavy chain (MyHC) expression was used as a marker for commitment to myogenic differentiation. (C) Myotube formation was determined for differentiating wild-type or emerin-null cells. Cells were considered differentiated myotubes if they contained >3 nuclei and were MyHC-positive. Values are mean±s.d.; *P<0.05; **P<0.01; ****P<0.001 using paired, two-tailed *t*-tests.

Cell cycle withdrawal, myosin heavy chain (MyHC) expression and the differentiation index (number of cells with >3 nuclei that were positive for MyHC) were monitored every 24 h for 72 h. After 24 h, more than 90% of wild-type progenitors withdrew from the cell cycle, whereas 16.7% of emerin-null myogenic progenitors were still in the cell cycle (*P*<0.01, Fig. 1). All wild-type progenitors withdrew from the cell cycle by 48 h, while 2.8% and 1.5% of emerin-null progenitors were still dividing after 48 and 72 h, respectively (Fig. 1, *P*<0.01). Myosin heavy chain was expressed in only 66.0% and 74.8% of differentiating emerin-null cells compared with 71.5% and 85.6% of differentiating wild-type progenitors at 48 h and 72 h, respectively (*P*<0.01). Differentiating emerin-null progenitors also failed to fuse and form mature myotubes as effectively as wild-type progenitors, as only 35.5% of emerin-null myotubes were formed after 72 h compared with 48.4% of wild-type cells (*P*<0.01). Collectively, these data show that emerin-null myogenic progenitors have impaired myogenic differentiation. The best time point to analyze changes in cell cycle withdrawal, myosin heavy chain expression and the differentiation index was determined to be 36 h after the induction of differentiation.

### Histone deacetylase activity regulates myogenic differentiation

**Fig. 2.**
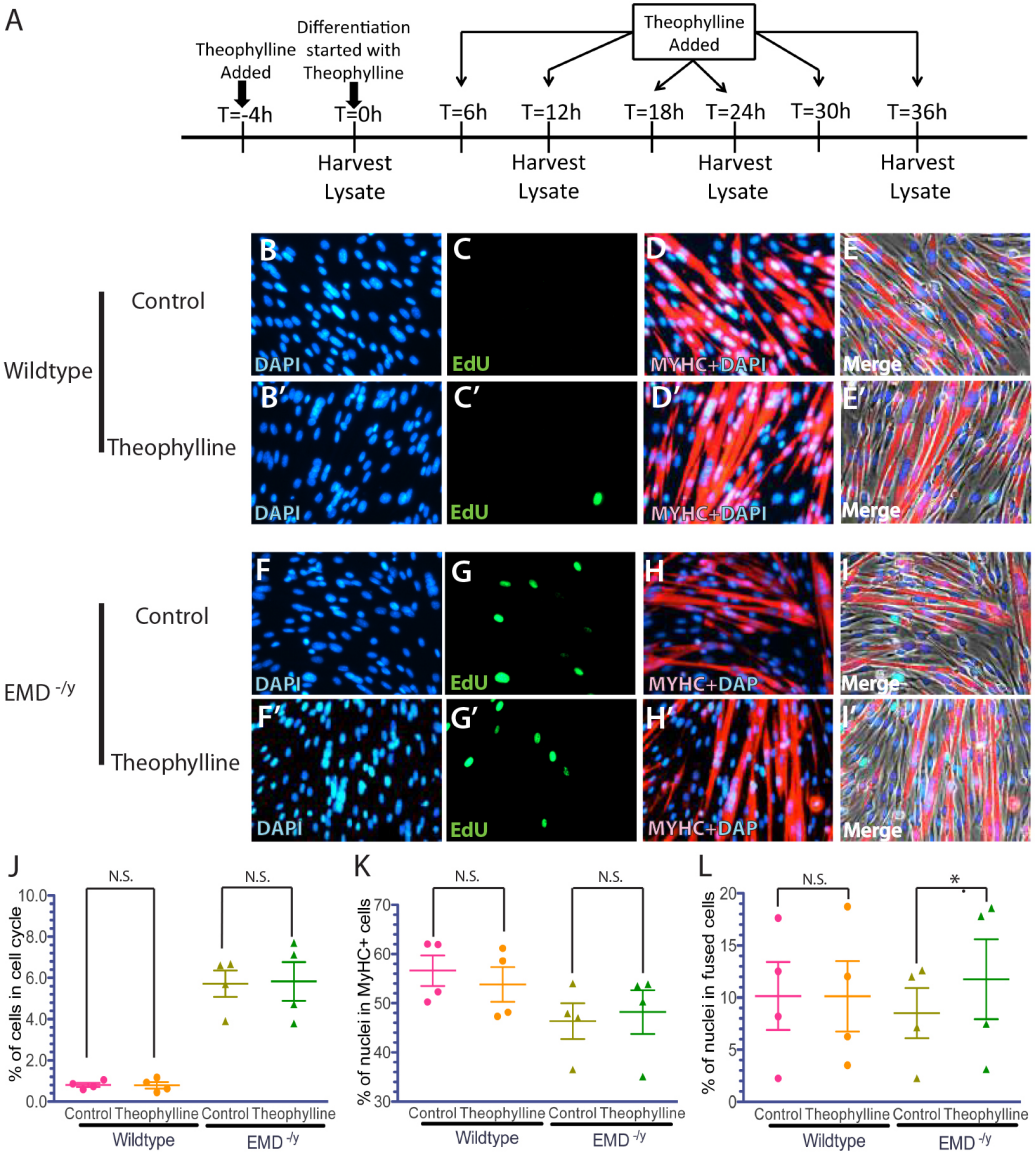
**Activation of HDAC3 activity by theophylline treatment rescues myotube formation in emerin-null myogenic progenitors.** (A) Timeline showing the timing of Theophylline addition and sample collection for western blot analysis of whole cell lysates during differentiation. (B-I′) Representative images of vehicle-treated wild-type (B-E) or emerin-null (F-I) cells and theophylline-treated wild-type (B′-E′) or emerin-null (F′-I′) cells 36 h after differentiation induction. 40× magnification. (J-L) Quantification of >1000 nuclei for each experimental treatment (*n*≥3) was carried out to determine the percentage of myogenic progenitors in the cell cycle (J), expressing MyHC (K) and that successfully formed myotubes (L) 36 h post-induction of differentiation. Results are mean±s.d. of *n*≥3; N.S., not significant; *P<0.05 using paired, two-tailed *t*-tests.

Theophylline treatment stimulated HDAC3 activity and rescued the localization of myogenic gene loci and their temporal expression during differentiation of emerin-null progenitors (Demmerle et al., 2013). To test whether HDAC3 activation rescued myogenic differentiation of emerin-null progenitors, emerin-null and wild-type myogenic progenitors were pretreated with 10 µM theophylline for 4 h prior to differentiation, followed by treatment with theophylline every 6 h for 36 h after induction of differentiation (Fig. 2A). Theophylline-treated wild-type progenitors exited the cell cycle normally. Emerin-null progenitors treated with theophylline had similar numbers of cells (6.0%) in the cell cycle as control emerin-null cells (5.6%; Fig. 2C,G,J). Theophylline treatment failed to rescue expression of MyHC in differentiating emerin-null progenitors, as 46.4% of control emerin-null cells and 48.2% of theophylline-treated emerin-null cells were MyHC-positive (Fig. 2D,H,K). Theophylline treatment rescued myogenic progenitor fusion during differentiation of emerin-null progenitors by 42% (12.0% treated versus 8.50% control; *P*=0.015; Fig. 2I,L). Wild-type progenitor fusion was unchanged by theophylline treatment (Fig. 2E,L). These results suggest HDAC3 acts during the latter stages of myogenic differentiation.

**Fig. 3.**
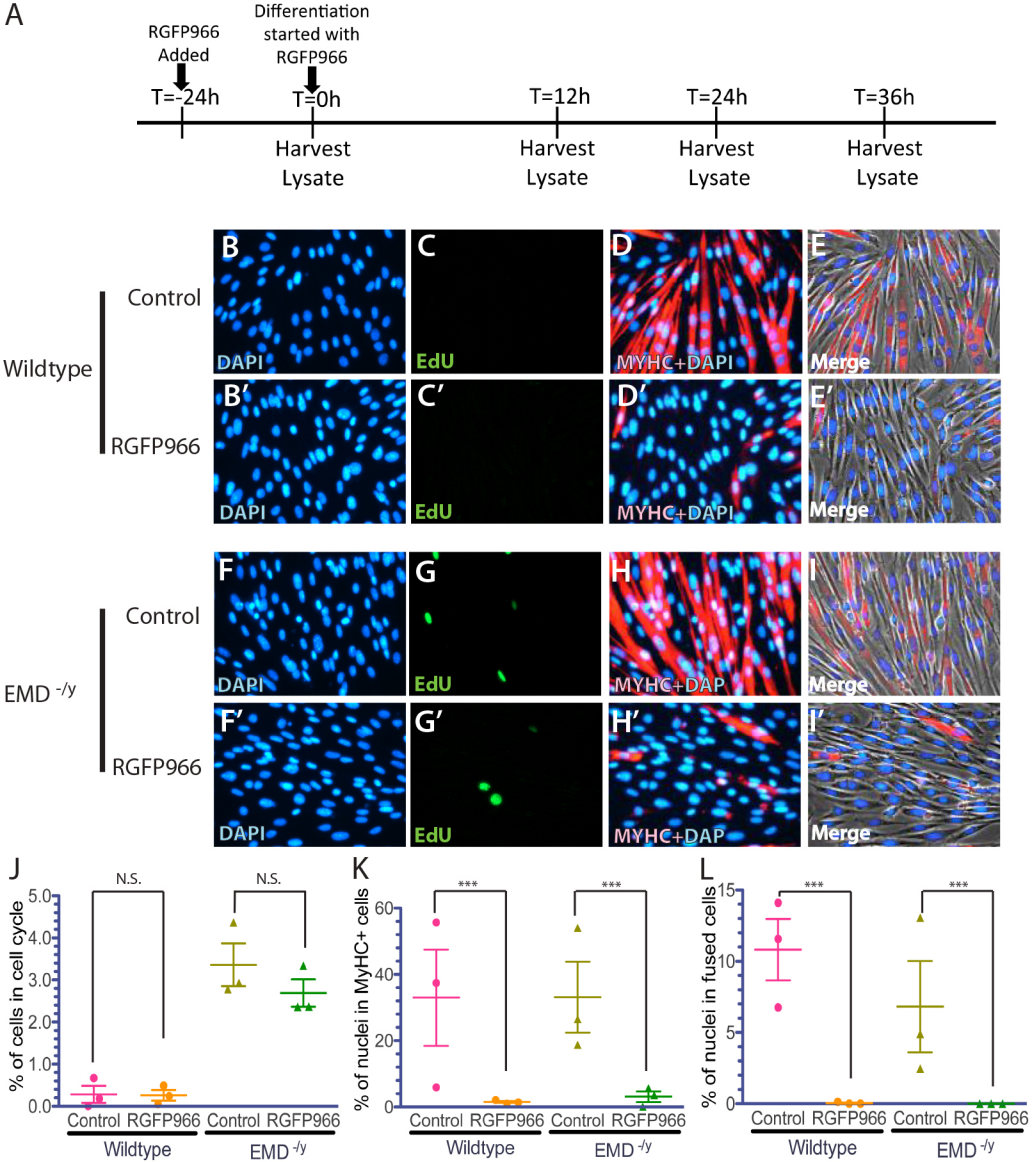
**HDAC3 inhibition by treatment with RGFP966 blocks MyHC expression and myotube formation during myogenic differentiation.** (A) Timeline showing the timing of RGFP966 addition and sample collection for western blot analysis of whole cell lysates during differentiation. (B-I′) Representative images of vehicle-treated wild-type (B-E) or emerin-null (F-I) cells and RGFP966-treated wild-type (B′-E′) or emerin-null (F′-I′) cells 36 h after differentiation induction. 40× magnification. (J-L) Quantification of >1000 nuclei for each experimental treatment (*n*≥3) was done to determine the percentage of myogenic progenitors in the cell cycle (J), are expressing MyHC (K) and formed myotubes (L) 36 h post-differentiation induction. Results are mean±s.d. of *n*≥3; N.S., not significant; ***P<0.001 using paired, two-tailed *t*-tests.

To independently confirm that HDAC3 activity is important for myogenic differentiation, wild-type and emerin-null myogenic progenitors were differentiated in the presence of the HDAC3-specific inhibitor RGFP966 (10 µM in DMSO) for 24 h prior to differentiation induction (Fig. 3A). HDAC3 inhibition had no effect on the withdrawal of wild-type myogenic progenitors from the cell cycle, as 0.423±0.32% of untreated progenitors and 0.264±0.283% (mean±s.d.) of RGFP966-treated progenitors were EdU-positive (Fig. 3C,J). RGFP966 treatment of emerin-null progenitors showed a small, but insignificant increase in cell cycle exit (2.69±0.729%) compared with untreated emerin-null cells (3.36±1.121%; Fig. 3G,J). Expression of myosin heavy chain was almost completely inhibited by RGFP966 treatment in both wild-type and emerin-null progenitors, as myosin heavy chain was expressed in only 1.51±0.913% and 3.1±2.59% of wild-type and emerin-null cells treated with RGFP966, respectively (Fig. 3D,H,K). Differentiation was completely inhibited, as only 0.04±0.129% and 0% of RGFP966-treated wild-type cells and emerin-null cells fused to form myotubes, respectively (Fig. 3D,E,H,I,L). Both wild-type and emerin-null cells align and elongate similar to untreated progenitors during the initial stages of differentiation, but both wild-type (Fig. 3D,E) and emerin-null (Fig. 3H,I) cells failed to pack tightly and fuse into myotubes. Instead they remained as individual cells.

**Fig. 4.**
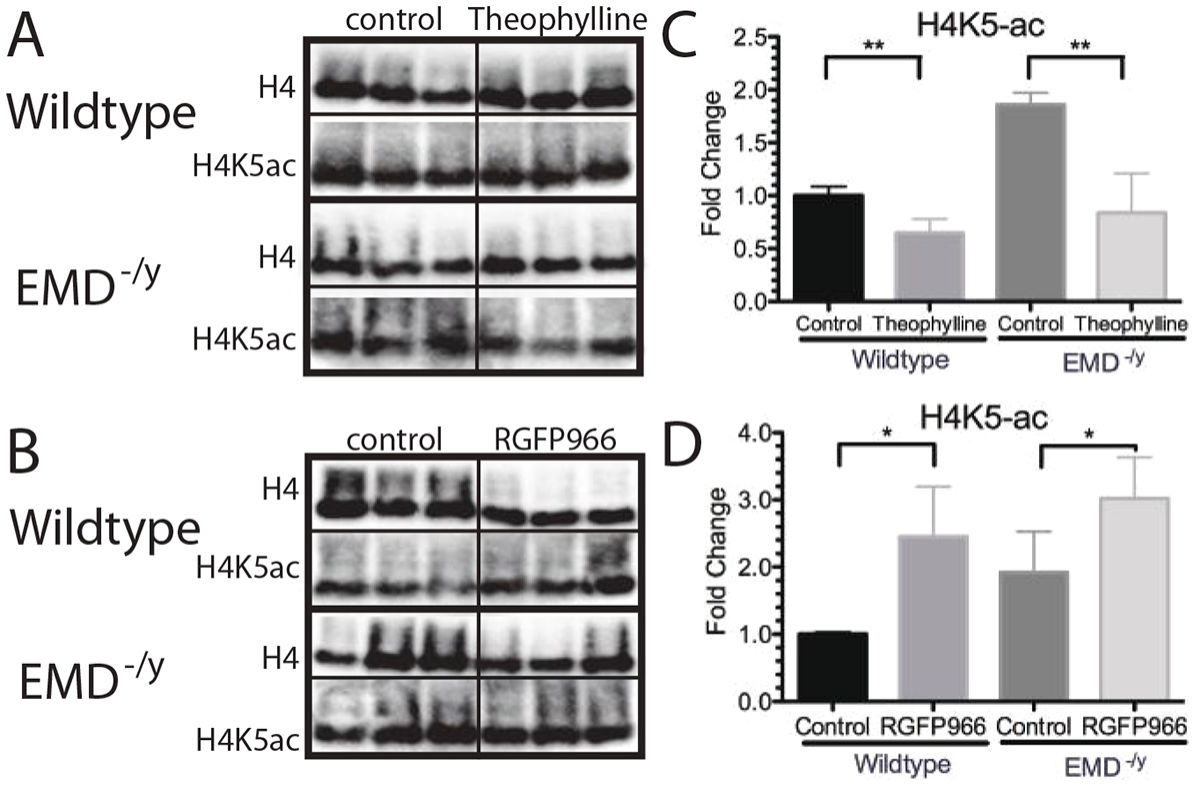
**H4K5 acetylation is reduced by activation of HDAC3 activity using theophylline and H4K5 acetylation increased by HDAC3 inhibition with RGFP966 treatment in myogenic progenitors.** H4K5ac is the main target of HDAC3 and was used to determine HDAC3 activity in cells. (A,B) Western blotting of whole cell lysates treated with theophylline (A) or RGFP966 (B) to analyze H4K5 acetylation in differentiating wild-type or emerin-null progenitors. Three biological replicates are shown for each treatment. (C,D) Densitometry was performed and H4K5ac protein levels in each sample were normalized to total H4 levels in each sample. Levels of H4K5ac in each treatment condition were then normalized to DMSO-treated wild-type cells. Results are mean±s.d. of *n*=3 for each condition; *P<0.05, **P<0.01 using unpaired, two-tailed *t*-tests.

Western blotting using antibodies against H4 and H4 acetylated on lysine 5 (H4K5ac) was used to confirm theophylline activated HDAC3 activity and RGFP966 repressed HDAC3 activity, respectively. Emerin-null cells increased levels of H4K5ac 1.9-fold, as expected (Fig. 4A-D). Treatment with theophylline caused a 35% reduction in H4K5ac in wild-type myogenic progenitors (Fig. 4A,C). Emerin-null progenitors treated with theophylline reduced H4K5ac levels by 54.9%, which resulted in a 26.2% decrease in H4K5ac compared with untreated wild-type myogenic progenitors. RGFP966 treatment increased H4K5ac 1.57-fold or 2.45-fold in emerin-null or wild-type myogenic progenitors, respectively (Fig. 4B,D). Increased H4K5ac by RGFP966 in emerin-null myogenic progenitors equates to a 3.02-fold increase in H4K5ac compared with levels in wild-type myogenic progenitors.

### ERK inhibition partially rescued emerin-null progenitor differentiation

**Fig. 5.**
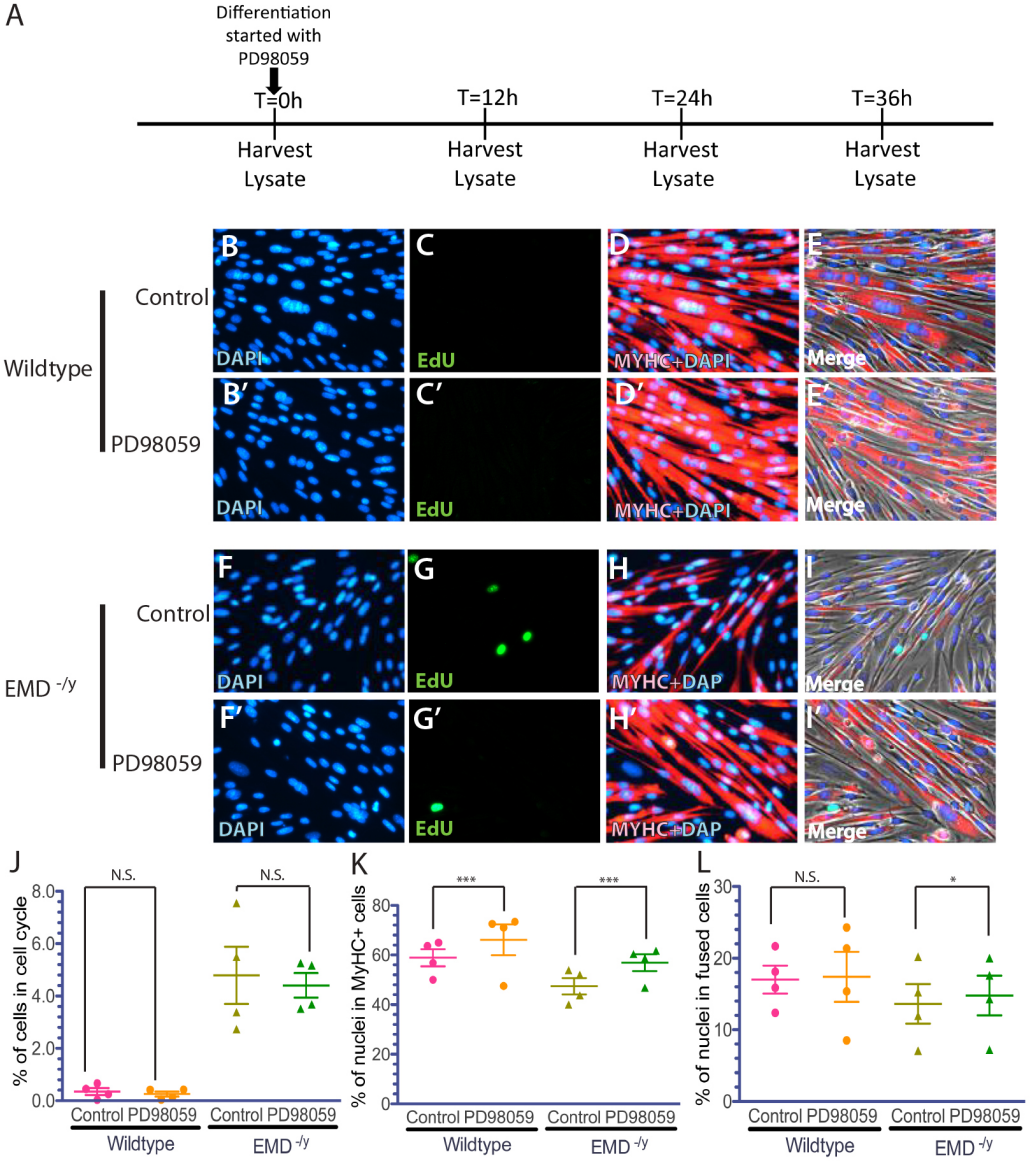
**ERK inhibition by PD98059 rescued MyHC expression and myotube formation during differentiation of emerin-null myogenic progenitors.** (A) Timeline showing the timing of PD98059 addition and sample collection for western blot analysis of whole cell lysates during differentiation. (B-I′) Representative images of vehicle-treated wild-type (B-E) or emerin-null (F-I) cells and PD98059-treated wild-type (B′-E′) or emerin-null (F′-I′) cells 36 h after differentiation induction. 40× magnification. (J-L) Quantification of >1000 nuclei for each experimental treatment (*n*≥3) was done to determine the percentage of myogenic progenitors in the cell cycle (J), that are expressing MyHC (K), and formed myotubes (L) 36 h post-differentiation induction. Results are mean±s.d. of *n*≥3; N.S., not significant; *P<0.05, ***P<0.001 using paired, two-tailed *t*-tests.

Previous research suggested that the ERK pathway might be important for the EDMD disease mechanism. Wild-type and emerin-null myogenic progenitors were incubated with U0126 or PD908059 to test if ERK inhibition rescues differentiation of emerin-null myogenic progenitors. PD98059 (10 µM) was added to myogenic progenitors upon differentiation induction and was present throughout differentiation (Fig. 5A); U0126 (10 µM) was added to wild-type or emerin-null progenitors for 1 h prior to differentiation induction and another hour after differentiation induction. DMSO was used as a negative control. These conditions were similar to those used previously to test ERK inhibition in C2C12 myoblasts (Favreau et al., 2008; Huber et al., 2009).

Cell cycle exit was not significantly improved in emerin-null progenitors treated with PD98059 (4.41±1.72% in treated emerin-null cells versus 4.79±2.09% in untreated emerin-null cells; Fig. 5G,J). The percentage of differentiating wild-type progenitors expressing MyHC increased from 58.9±2.27% to 66.1±3.86% upon treatment with PD98059 (*P*<0.01; Fig. 5D,E,K) as anticipated (Favreau et al., 2008). The percentage of emerin-null progenitors expressing MyHC increased from 49.9±6.43% to 56.9±6.81% upon treatment with PD98059 (Fig. 5H,I,K; *P*<0.05). Myotube formation was increased from 13.0±4.80% in differentiating emerin-null progenitors to 15.4±4.73% in PD98059-treated differentiating emerin-null progenitors (*P*<0.01; Fig. 5H,I,L). There was no significant difference in myotube formation of untreated or PD98059-treated wild-type myogenic progenitors (Fig. 4D,E,L).

**Fig. 6.**
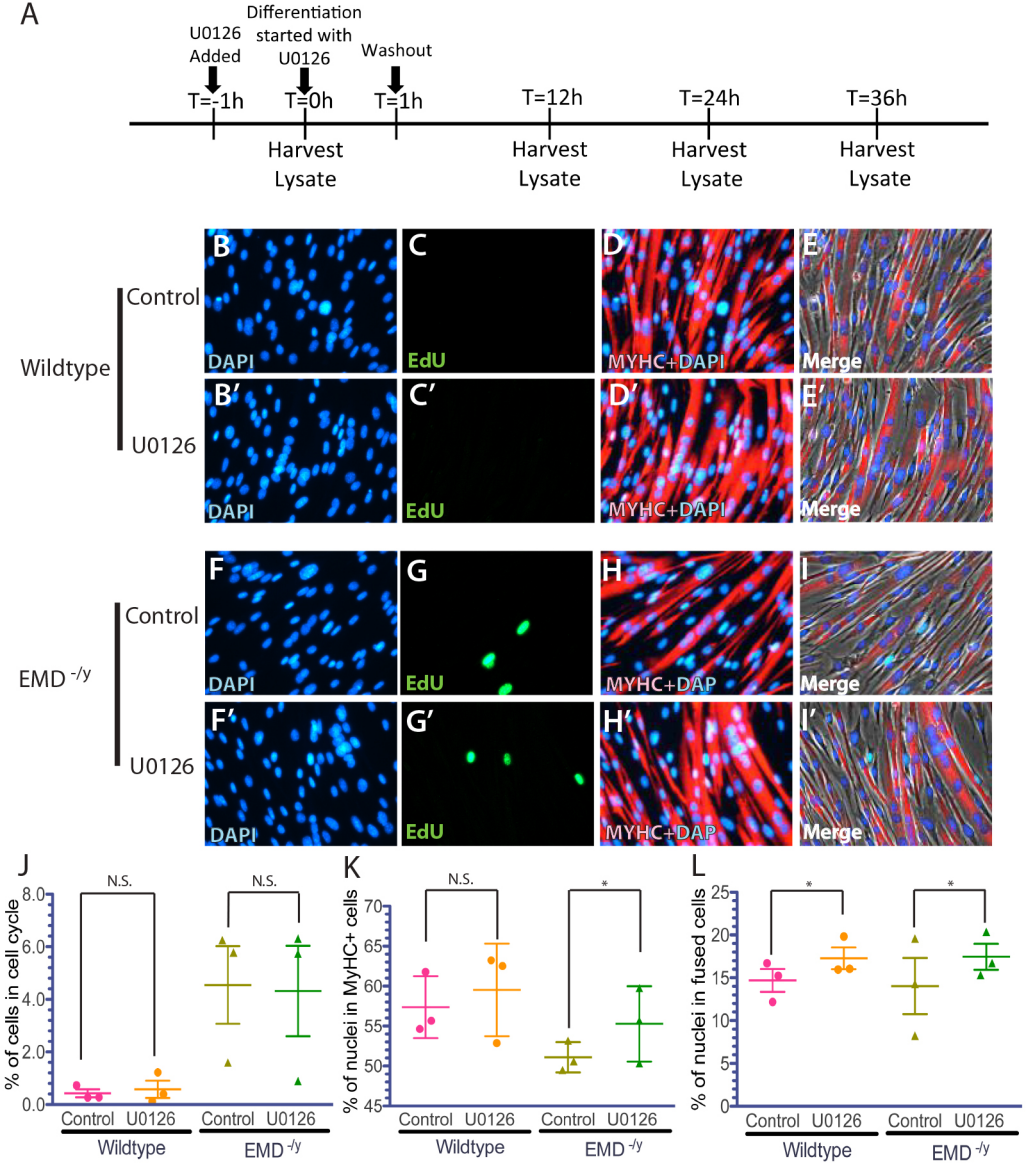
**ERK inhibition by U0126 treatment rescued MyHC expression and myotube formation in differentiating emerin-null progenitors.** (A) Timeline showing the timing of U0126 addition and sample collection for western blot analysis of whole cell lysates during differentiation. (B-I′) Representative images of vehicle-treated wild-type (B-E) or emerin-null (F-I) cells and U0126-treated wild-type (B′-E′) or emerin-null (F′-I′) cells 36 h after differentiation induction. 40× magnification. (J-L) Quantification of >1000 nuclei for each experimental treatment (*n*≥3) was done to determine the percentage of myogenic progenitors in the cell cycle (J), percentage of cells expressing MyHC (K) and the number of myotubes formed (L) 36 h post-differentiation induction. Results are mean±s.d. of *n*≥3; N.S., not significant; *P<0.05 using paired, two-tailed *t*-tests.

Cell cycle exit was slightly improved in differentiating emerin-null progenitors treated with U0126 (4.14±2.65% in emerin-null cells versus 4.54±2.5% in untreated cells; Fig. 6A-C, G,J). Treatment with U0126 had no effect on wild-type progenitor cell cycle exit. The percentage of emerin-null progenitors expressing MyHC increased from 51.09±3.87% in mock-treated cells to 55.27±4.31% in U0126-treated cells (*P*<0.05; Fig. 6H,I,K). There was no change in the percentage of MyHC-positive wild-type progenitors differentiated for 36 h (Fig. 6D,E,K). Myotube fusion also increased from 14.0±6.00 in differentiating emerin-null progenitors to 17.54±3.0% in U0126-treated emerin-null cells, which are levels similar to that seen in untreated wild-type cells (*P*<0.05; Fig. 6D,E,H,I,L). Collectively, these results show that MyHC expression and myotube formation are rescued during differentiation of emerin-null myogenic progenitors by inhibiting ERK activity.

**Fig. 7.**
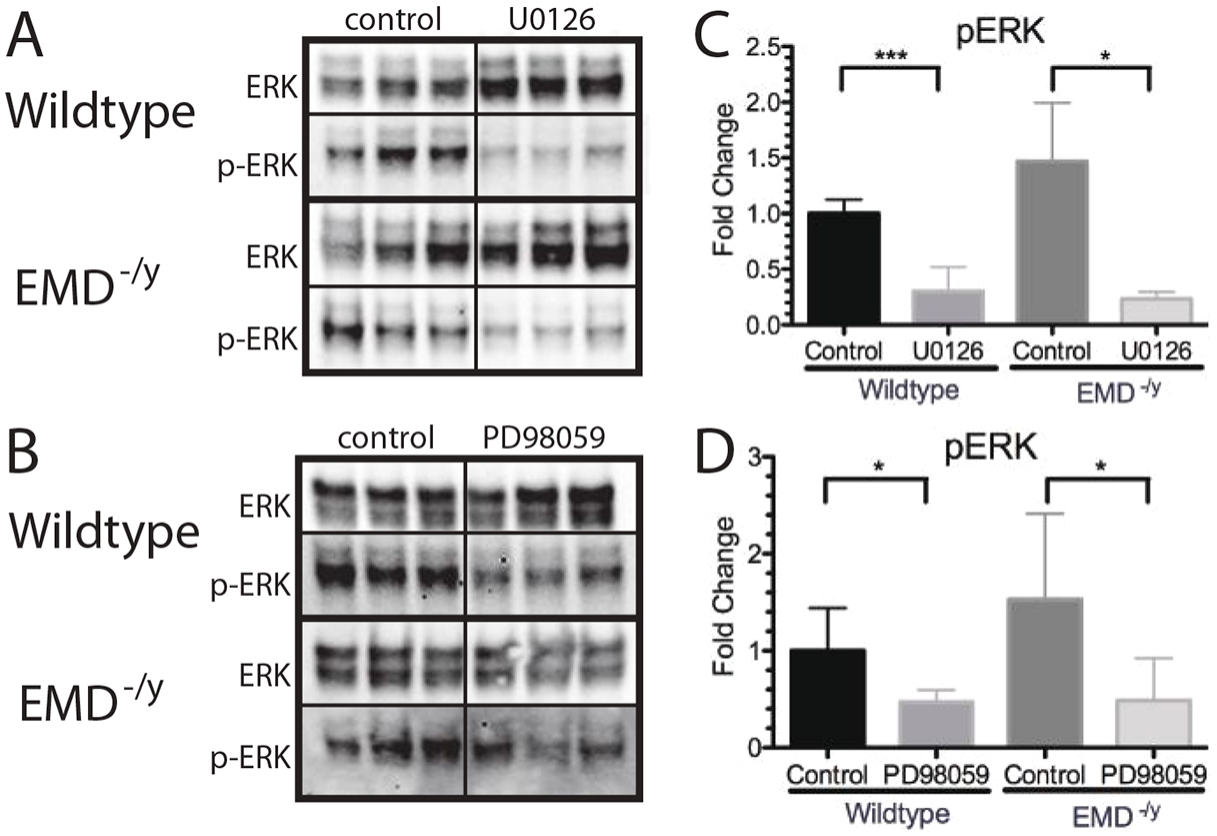
**ERK phosphorylation is decreased by treatment with the ERK inhibitors U0126 and PD98059 in differentiating myogenic progenitors.** (A,B) Western blotting of whole cell lysates treated with U0126 (A) or PD98059 (B) to analyze ERK activation during differentiation of wild-type or emerin-null progenitors. DMSO-only treatment was the control. Three biological replicates are shown for each treatment. (C,D) Densitometry was performed and phosphorylated ERK in each sample was normalized to total ERK protein in each sample. Levels of phosphorylated ERK for each condition were normalized to DMSO-treated wild-type cells. Results are mean±s.d. of *n*=3 for each condition; *P<0.05, ***P<0.005 using unpaired, two-tailed *t*-tests.

Western blotting using antibodies against ERK and phosphorylated ERK (p-ERK) was done to confirm inhibition of ERK activation by treatment with PD98059 or U0126. Emerin-null cells increased p-ERK 1.5-fold compared with wild-type myogenic progenitors, as expected (Fig. 7A-D). Treatment with U0126 caused a 70% reduction in p-ERK in wild-type myogenic progenitors (Fig. 7A,C). Emerin-null progenitors treated with U0126 reduced p-ERK levels by 77.6%, which resulted in a 65.5% decrease in p-ERK as compared with untreated wildtype myogenic progenitors. PD98059 treatment reduced p-ERK by 51.1% or 52.3% in emerin-null or wild-type myogenic progenitors, respectively (Fig. 7B,D). Reduction in p-ERK by PD98059 in emerin-null myogenic progenitors equates to 74.6% of wild-type p-ERK levels.

### p38 MAPK inhibition impairs myogenic differentiation

**Fig. 8.**
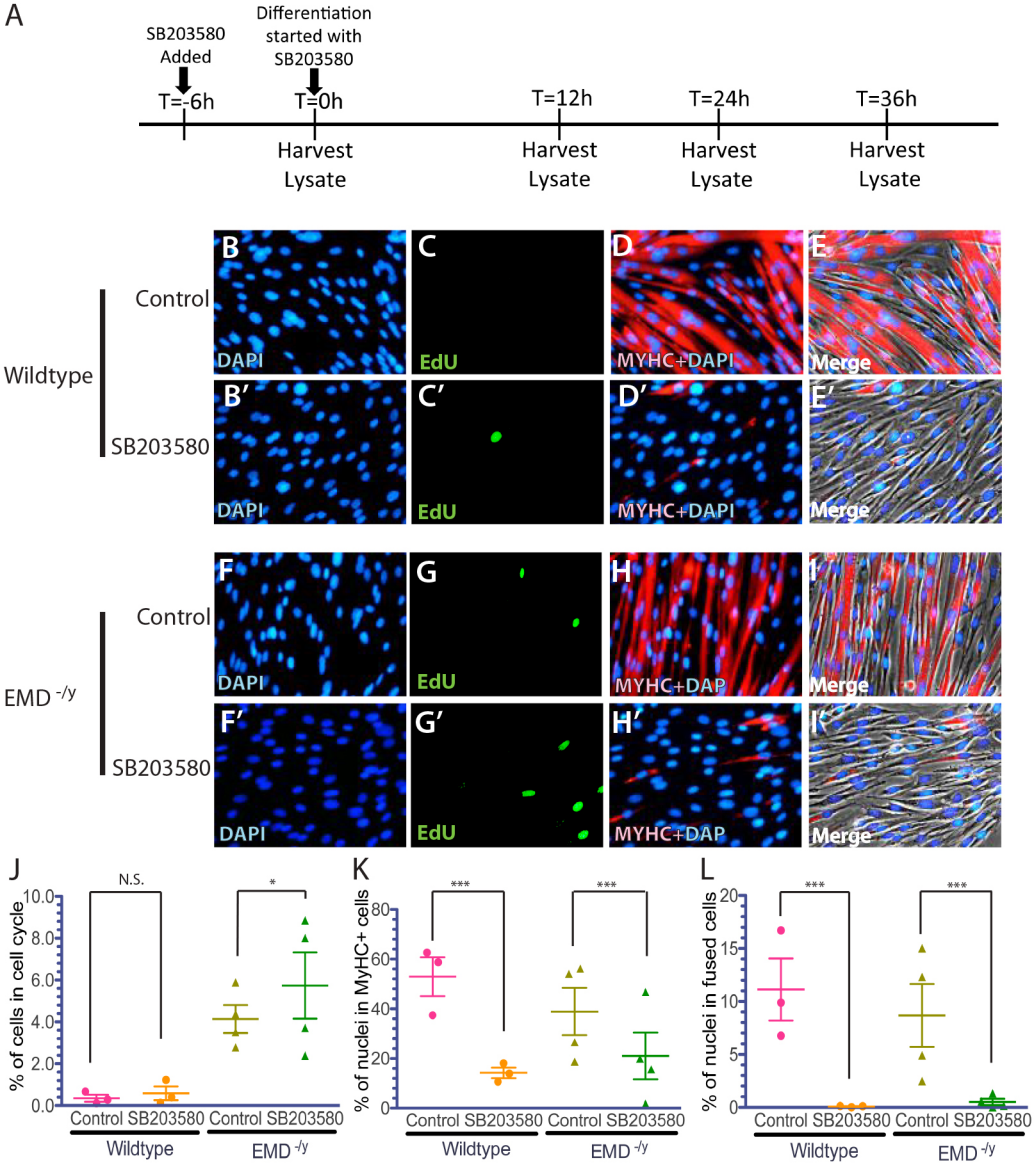
**p38 MAPK inhibition by SB203580 treatment prevents myogenic differentiation in wild-type and emerin-null myogenic progenitors.** (A) Timeline showing the timing of SB203580 addition and sample collection for western blot analysis of whole cell lysates during differentiation. (B-I′) Representative images of vehicle-treated wild-type (B-E) or emerin-null (F-I) cells and SB203580-treated wild-type (B′-E′) or emerin-null (F′-I′) cells 36 h after differentiation induction. 40× magnification. (J-L) Quantification of >1000 nuclei for each experimental treatment (*n*≥3) was done to determine the percentage of myogenic progenitors in the cell cycle (J), percentage of cells expressing MyHC (K) and the number of myotubes formed (L) 36 h post-differentiation induction. Results are mean±s.d. of *n*≥3; N.S., not significant; *P<0.05, ***P<0.001 using paired, two-tailed *t*-tests.

The p38 MAPK-specific inhibitor SB203580 was added at 10 µM to wild-type or emerin-null myogenic progenitors 6 h prior to differentiation and was incubated with the cells throughout differentiation to test if inhibition of p38 MAPK rescued emerin-null myogenic differentiation. The percentage of wild-type myogenic progenitors exiting the cell cycle decreased 1.75-fold by treatment with SB203580 (Fig. 8C,J). Cell cycle exit was also inhibited in SB203580-treated differentiating emerin-null progenitors, as the number of EdU-positive cells increased 1.64-fold (Fig. 8G,J). SB203580 treatment of differentiating wild-type myogenic progenitors significantly decreased the number of cells expressing MyHC by 3.73-fold, as only 14.2% of wild-type progenitors were MyHC-positive (Fig. 8D,E,K); 20.9% of SB203580-treated emerin-null myogenic progenitors were MyHC-positive (Fig. 8G,H,K), representing a 1.87-fold decrease in MyHC-expressing emerin-null cells. Vehicle-treated emerin-null progenitors showed a 1.4-fold reduction in MyHC-expressing cells. Myotube formation was significantly inhibited in differentiating wild-type and emerin-null progenitors. Only 0.0789% of SB203580-treated wild-type myogenic progenitors formed myotubes, compared with 11.1% of vehicle-treated controls (Fig. 8D,E,L). Similarly, treatment of differentiating emerin-null myogenic progenitors with SB203580 resulted in a 17.1-fold decrease in the number of myotubes (Fig. 8H,I,L). Thus, treatment of emerin-null myogenic progenitors with SB203580 fails to rescue their impaired differentiation. Rather, inhibition of the p38 MAPK pathway impaired the earliest steps of myogenic differentiation, including myogenic progenitor commitment to differentiation and myotube formation.

**Fig. 9.**
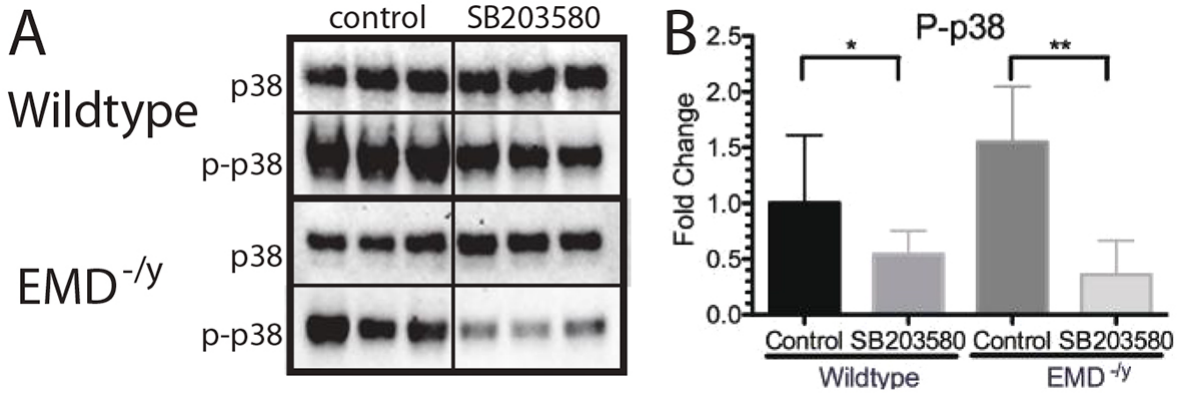
**p38 MAPK phosphorylation is decreased by treatment with the p38 MAPK inhibitor SB203580 in differentiating myogenic progenitors.** (A) Western blotting of whole cell lysates treated with SB203580 was performed to analyze activation of p38 MAPK during differentiation of wild-type or emerin-null progenitors. DMSO treatment was the control. Three biological replicates are shown for each treatment. (B) Densitometry was performed and phosphorylated p38 MAPK was normalized to total p38 MAPK protein in each sample. Levels of phosphorylated p38 MAPK for each condition were normalized to DMSO-treated wild-type cells. Results are mean±s.d. of *n*=3 for each condition; *P<0.05, **P<0.01 using unpaired, two-tailed *t*-tests.

Western blotting using antibodies against p38 MAPK and phosphorylated p38 MAPK (p-p38 MAPK) was used to confirm treatment with SB203580 inhibited p38 MAPK phosphorylation. p38 MAPK was activated in emerin-null cells, as anticipated (Koch and Holaska, 2012) with a 1.55-fold increase in p-p38 MAPK (Fig. 9A,B). Treatment with SB203580 caused a 45.7% reduction in p-p38 MAPK in wild-type myogenic progenitors (Fig. 9A,B). Emerin-null progenitors treated with SB203580 reduced p-p38 MAPK levels by 76.8%, which resulted in a 66.0% decrease in p-p38 MAPK compared with levels in vehicle-treated wild-type myogenic progenitors.

## DISCUSSION

Multiple lines of evidence support the hypothesis that the skeletal muscle pathology of EDMD is caused, at least in part, by inefficient skeletal muscle regeneration. Emerin-null mice exhibit motor coordination defects and delayed skeletal muscle regeneration and repair (Melcon et al., 2006; Ozawa et al., 2006). Emerin-null myoblasts and emerin-downregulated myoblasts also exhibit impaired differentiation (Frock et al., 2006; Huber et al., 2009). Increased skeletal muscle damage is seldom seen in EDMD patients (e.g. no increased skeletal muscle fiber permeability) but skeletal muscle biopsies from EDMD patients and emerin-null mice have increased expression of genes important for skeletal muscle regeneration (Bakay et al., 2006; Melcon et al., 2006). In emerin-null myogenic progenitors, the signaling pathways important for myogenic differentiation and skeletal muscle regeneration (Conboy and Rando, 2002; Edwall et al., 1989; Jennische and Hansson, 1987; Koch and Holaska, 2012; Massague et al., 1986; Polesskaya et al., 2003; Ridgeway et al., 2000) as well as the coordinated temporal expression of genes involved in myogenic differentiation (*Myod1*, *Myf5*, *Pax3* and *Pax7*; Demmerle et al., 2013) are disrupted.

**Fig. 10.**
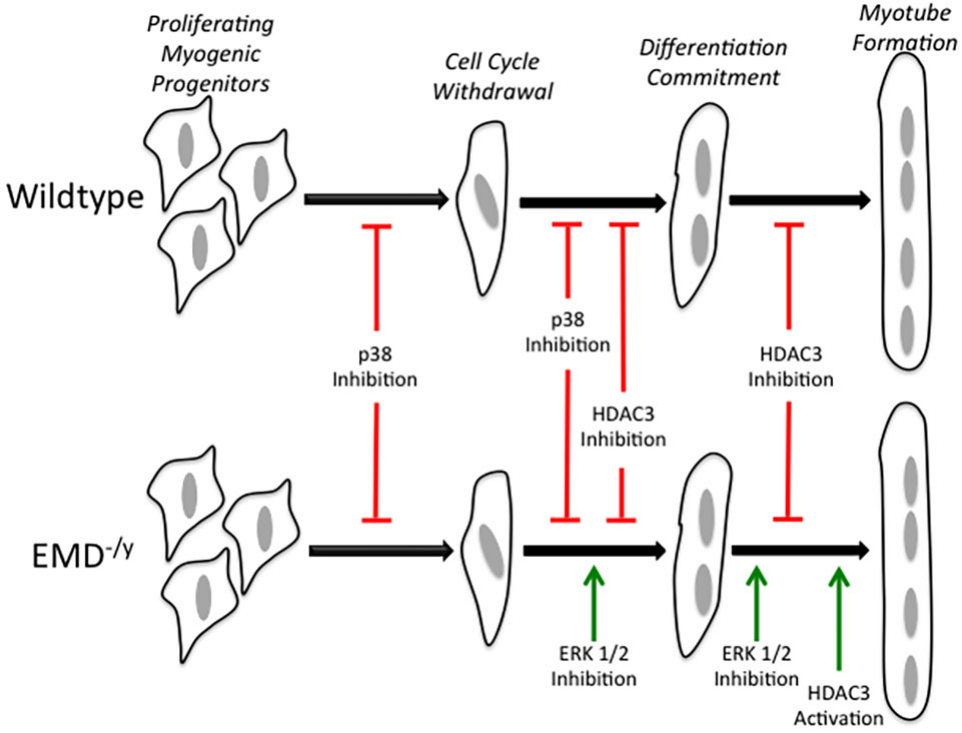
**ERK, p38 MAPK and HDAC3 regulate specific transition stages during myogenic differentiation.** The stages of myogenic differentiation in wild-type (top) and emerin-null (bottom) myogenic progenitors are illustrated. Inhibition of p38 MAPK activity blocks cell cycle withdrawal and commitment to myogenic differentiation in both wild-type and emerin-null progenitors. HDAC3 inhibition blocks differentiation commitment and myotube formation in both wild-type and emerin-null progenitors. ERK inhibition rescues differentiation commitment and myotube formation in emerin-null progenitors with no effect on wild-type differentiation. Activation of HDAC3 catalytic activity rescues myotube formation in emerin-null myogenic progenitors with no effect on wild-type differentiation. Green arrows indicate rescue; red lines indicate blockade of differentiation progression.

Emerin is proposed to play an important role in the regulation of myogenic differentiation by two potential mechanisms: the regulation of chromatin architecture and the regulation of intracellular signaling cascades. The results presented here show that activation of p38 MAPK and ERK is disrupted during differentiation of pure populations of bona-fide emerin-null myogenic progenitors. Rescue of phosphorylated ERK to wild-type levels using two different ERK inhibitors partially rescued emerin-null myogenic differentiation. Thus, misregulation of the ERK pathway is likely to contribute to the mechanism underlying the impaired differentiation of emerin-null myogenic progenitors. Our studies also define the specific stages at which p38 MAPK and ERK function during myogenic differentiation and demonstrate the importance of HDAC3 in regulating myogenic differentiation in two different ways. First, activation of HDAC3 rescues emerin-null myotube formation with no significant rescue of cell cycle withdrawal or MyHC expression. Second, inhibition of HDAC3 reduced cell cycle withdrawal, decreased MyHC expression and decreased myotube formation. How the ERK, p38 MAPK and HDAC3 pathways function in myogenic differentiation is summarized in Fig. 10.

### ERK and p38 MAPK signaling are required for myogenic differentiation

Emerin-null and lamin A mutant cells were previously shown to activate ERK signaling (Muchir et al., 2007a,b), which is predicted to contribute to EDMD pathology. Transient inhibition of ERK with PD98059 during the first two days of differentiation in C2C12 cells expressing *LMNA*-R453W mutants rescued myotube formation by day 6 (Favreau et al., 2008). Another study showed transient ERK inhibition in emerin-knockdown cells by U0126-rescued differentiation after 4 days (Huber et al., 2009). However, the myogenic index used in this study only counted the number of nuclei in MyHC-positive cells, not the number of nuclei in fused cells, as was done in our study. In our study, treatment with ERK inhibitors rescued MyHC expression and myotube formation in a purified population of bona-fide emerin-null myogenic progenitors. These results confirm and extend previous results with emerin-downregulated C2C12 myoblasts (Favreau et al., 2008; Huber et al., 2009; Muchir et al., 2007a,b).

The exact mechanism responsible for activation of ERK signaling during myogenic differentiation of emerin-null progenitors is not known. Our emerin-null myogenic progenitors are an ideal system to interrogate emerin regulation of the ERK pathway during differentiation. Myogenic cells experience biphasic ERK activation, where ERK signaling is necessary for satellite cell proliferation and myotube formation (Bennett and Tonks, 1997; Cho et al., 2007; Coolican et al., 1997; Wu et al., 2000; Yang et al., 2006; Yokoyama et al., 2007). ERK inhibition is required early during differentiation to initiate myogenesis and early and late ERK activity is crucial for proper myogenic differentiation (Jo et al., 2009; Li et al., 2000; Rommel et al., 1999; Tiffin et al., 2004; Wu et al., 2000).

Reduced lamin A or emerin expression causes increased ERK activity and impairs myogenic differentiation (Muchir et al., 2009b) and treatment with ERK inhibitors rescues their myogenic differentiation (Favreau et al., 2008; Huber et al., 2009). ERK is also activated in the hearts of mice lacking emerin expression (Muchir et al., 2007a) and in *Lmna*^H222P/H222P^ mice, which are a model of EDMD2 (Muchir et al., 2009a, 2014; Muchir and Worman, 2016; Wu et al., 2011).

Transient ERK inhibition can prevent dilated cardiomyopathy in lamin A mutant mice through the TGF-β/SMAD signaling axis (Chatzifrangkeskou et al., 2016). TGF-β acts early during differentiation to prevent activation of myogenic genes and antagonizes fusion of myocytes to myotubes (Massague et al., 1986; Olson et al., 1986) and addition of TGF-β1 to C2C12 myoblasts blocks IGF signaling to inhibit muscle differentiation and muscle repair (Gardner et al., 2011). Reduced expression of myogenic differentiation factors correlates with the increased expression of myogenic progenitor proliferation factors in myoblasts treated with TGF-β (Schabort et al., 2009). We previously showed that emerin-null myogenic progenitors have decreased levels of TGF-β (Koch and Holaska, 2012). Thus, we predict that the TGF-β/SMAD signaling axis likely plays an important role in the altered ERK signaling seen during differentiation of emerin-null myogenic cells. Whether emerin functionally interacts with TGF-β and ERK signaling to regulate both satellite cell activation and myotube formation remains to be seen.

ERK and p38 MAPK signaling complement one another and together promote proper myogenic differentiation (Wu et al., 2000). Transient ERK inhibition in concert with p38 MAPK activation is required for controlling the coordinated temporal expression of differentiation genes during myogenic differentiation (Segales et al., 2016). p38 MAPK is an indispensable *Myod1* activator (Hausburg et al., 2015; Jones et al., 2005) and sustained levels of p38 MAPK are required for the formation of MyHC-positive myotubes (Wu et al., 2000). Additionally, myogenic differentiation is accelerated in myoblasts expressing constitutively active p38 MAPK. Emerin-null myogenic progenitors have increased levels of phosphorylated p38 MAPK (Koch and Holaska, 2012; this study) and inhibition of p38 MAPK arrests differentiation. Thus, maintaining the correct levels of phosphorylated p38 MAPK within a narrow range appears to be required for proper myogenic differentiation. A more nuanced experimental approach will be required to determine how emerin impacts the p38 MAPK pathway to regulate myogenic differentiation, including the interrogation of each pathway component for regulation by emerin. Furthermore, crosstalk between p38 MAPK, ERK and TGF-β pathways, as well as other MAPK pathways, will need to be examined.

### HDAC3 activity during myogenic differentiation

The genome is organized in a cell-type-specific manner to ensure a particular cell type expresses the proper repertoire of genes. The genome is dynamically reorganized during development and stem cell differentiation to regulate the coordinated temporal expression of differentiation genes. Typically, active genes localize to the nuclear interior and silenced genes preferentially localize to distinct subnuclear compartments, including the nuclear lamina and nucleoli (Kind et al., 2013; Kind and van Steensel, 2014; Mattout et al., 2015; Reddy et al., 2008; Zullo et al., 2012). Portions of the genome that interact with the nuclear lamina are called lamina-associated domains (LADs). LADs were initially defined by their association with A- and B-type lamins (Guelen et al., 2008; Pickersgill et al., 2006; Zullo et al., 2012). Recent evidence showed that lamins are not required for LAD formation (Amendola and van Steensel, 2015), suggesting that other nuclear envelope proteins mediate the interaction of LADs with the nuclear envelope. We predict that emerin is one of these proteins important for repressed chromatin interaction with the nuclear lamina at the nuclear periphery.

Growing evidence shows that emerin has a role in establishing, maintaining or recruiting repressed chromatin to the nuclear lamina at the nuclear envelope. Chromatin adopts a more relaxed chromatin configuration in emerin-null cells (Fidziańska and Hausmanowa-Petrusewicz, 2003; Mewborn et al., 2010; Ognibene et al., 1999). Emerin and LAP2β interact with chromatin regulatory complexes containing BAF or histone deacetylases (HDACs; Demmerle et al., 2012; Holaska and Wilson, 2007; Somech et al., 2005). Emerin binds directly to HDAC3, the catalytic component of the nuclear co-repressor (NCoR) complex (Demmerle et al., 2012; Holaska and Wilson, 2007). Binding of emerin to HDAC3 activates HDAC3 activity and recruits it to the nuclear envelope; this functional interaction coordinates the spatiotemporal nuclear envelope localization of genomic regions containing *Myf5*, *Myod1* and *Pax7* to ensure differentiation proceeds normally (Demmerle et al., 2012, 2013). Loss of emerin disrupts this genomic reorganization, which is rescued by treatment with theophylline. LAP2β also interacts with HDAC3 and induces H4 deacetylation (Somech et al., 2005) to contribute to LAD formation (Zullo et al., 2012). Thus, rescue of genomic organization and myogenic differentiation by theophylline in emerin-null cells likely results from an increase in the association of HDAC3 with LAP2β, which rescues the coordinated temporal sequestration and silencing of promoters to temporally regulate the differentiation transcriptional program.

In this study, myotube formation was rescued by theophylline treatment. Previous work showed that theophylline rescued genomic organization and the expression of differentiation genes (Demmerle et al., 2013). Here, HDAC3 activity was shown to be important for the latter steps of myogenic differentiation. This suggests that emerin regulation of HDAC3 activity might specifically control the coordinated temporal expression of genes important for cell fusion or myotube maturation. Alternatively, emerin regulation of HDAC3 activity may be important early during differentiation to coordinate the temporal expression of both early and late differentiation genes, but the defect is not apparent until later in differentiation. Consistent with these results, HDAC3 inhibition by RGFP966 blocks MyHC expression and fusion in both differentiating wild-type and emerin-null myogenic progenitors. We propose that HDAC3 activity is required for the transition from proliferating myogenic progenitors to differentiating myoblasts by repressing the expression of genes important for myogenic progenitor proliferation and induction of the differentiation gene program, since inhibition of HDAC3 blocks this transition. Furthermore, we propose that HDAC3 activity is also required for myotube formation, since HDAC3 activation rescued myotube formation in emerin-null progenitors.

Our results support the existence of crosstalk between HDAC3, p38 MAPK and ERK molecular pathways in the regulation of myogenic differentiation by emerin. Supporting this hypothesis, HDAC3 was recently shown to inhibit ERK expression and ERK phosphorylation (Carpio et al., 2016). These results in chondrocytes are consistent with our results during myogenic differentiation since this model posits that HDAC3 activation would inhibit ERK phosphorylation. We do see an inverse relationship between HDAC3 and ERK in myogenic progenitors, as emerin-null myogenic progenitors have increased HDAC3 activity (Demmerle et al., 2012, 2013) and decreased ERK phosphorylation. Further HDAC3 activation and ERK inhibition both rescue myogenic differentiation of emerin-null cells. The studies presented here also show that HDAC3 acts during two stages of myogenic differentiation, depending on whether HDAC3 is activated or inhibited. This is similar to the stages in which ERK acts. It will be interesting to determine whether HDAC3 activation inhibits ERK phosphorylation and whether HDAC3 inhibition increases ERK phosphorylation in our experimental system.

## MATERIALS AND METHODS

### Cell culture

Wild-type and emerin-null H2K myogenic progenitors were maintained as previously described (Cohen et al., 2013; Koch and Holaska, 2012). Proliferating wild-type and emerin-null H2K myogenic progenitors were seeded at ∼650 cells/cm^2^ onto tissue culture plates (CellStar by Greiner Bio-One) coated with 0.01% gelatin (Sigma-Aldrich) and maintained at 33°C and 10% CO_2_ in proliferative medium (high glucose DMEM supplemented with 20% heat-inactivated fetal bovine serum, 2% L-glutamine, 2% chick embryo extract, 1% penicillin/streptomycin, sodium pyruvate, 20 units/ml γ-interferon).

For myogenic differentiation 25,000 cells/cm^2^ were seeded into 12-well dishes (Greiner Bio-One) or 6-well dishes (Greiner Bio-One) coated with 0.01% gelatin and maintained in proliferative conditions for 24 h. Myogenic differentiation was stimulated by replacing proliferation medium with differentiation medium (high glucose DMEM with sodium pyruvate, 5% horse serum, 2% L-glutamine) and incubating the cells at 37°C and 5% CO_2_.

### Pharmacological treatments

Theophylline (Sigma-Aldrich) was dissolved in H_2_O to make a 1.0 mM stock solution, which was added to proliferation medium to make a final concentration of 10 µM theophylline. Theophylline or the appropriate volume of H_2_O was added to cells 4 h prior to differentiation, with substitution of the differentiation medium, followed by the addition of 10 µM theophylline every 6 h for 36 h.

RGFP966 (SelleckChem, NC0574889) was dissolved in DMSO to form a 10 mM stock solution and was added to the proliferation medium to a final concentration of 10 µM 24 h prior to differentiation and again with differentiation medium at *t*=0. DMSO alone was used as a control.

PD98059 (Life Technologies, PHZ1164) and U0126 (Cell Signaling Technology, 9903) were diluted to 10 mM in DMSO and the appropriate volume of each was added to reach a final concentration of 10 µM in proliferation or differentiation medium. The appropriate volume of PD98059 or DMSO alone was added to the differentiation medium at *t*=0 and to the proliferation medium 1 h prior to differentiation and again upon addition of differentiation media at *t*=0. Both U0126 and DMSO were removed after 1 h and fresh differentiation medium was added, as previously described (Huber et al., 2009).

SB203580 (Life Technologies, PHZ1253) was dissolved in DMSO to form a 10 mM stock solution. A final concentration of 10 µM SB203580 or the corresponding volume of DMSO alone was added to the proliferation medium 6 h prior to differentiation and to the differentiation medium at *t*=0.

### EdU incorporation and immunofluorescence microscopy

Proliferating or differentiating myogenic progenitors were treated with 10 µM EdU in DMSO and incubated for 2 h. The cells were then fixed with 3.7% formaldehyde in PBS for 15 min, washed three times with PBS, and stored at 4°C with 0.1% sodium azide in PBS until cells were processed as per the manufacturer's instructions (Life Technologies). The cells were permeabilized in 0.5% Triton X-100 in PBS for 20 min, washed three times with 3% BSA in PBS and treated with a Click-IT EdU reaction cocktail. Cells were blocked for 1 h at room temperature with 3% BSA in PBS containing 0.1% Triton X-100. For immunofluorescence microscopy, cells were stained with rabbit anti-MyHC antibody (1:20, Santa Cruz Biotechnologies, H-300), washed three times with PBS, and stained with an Alexa Fluor 594 goat anti-rabbit secondary antibody (1:200, Life Technologies, C10637). Nuclei were stained with DAPI and the cells were stored in PBS with 0.1% sodium azide until imaging.

Cells were imaged using the EVOS-FL imaging system (Life Technologies) using a long working distance 40× objective. Ten different sections of the well were used to obtain images. Each field had ∼100-200 cells per field and a total of 1000-2000 nuclei were analyzed for each experiment. Four different images for each field were obtained for this analysis: phase contrast, blue fluorescent channel (for DAPI stained nuclei), green fluorescent channel (for EdU positive nuclei) and red fluorescent channel (for MyHC). Nuclei and cells were counted using either the EVOS system or the cell counter plugin on ImageJ. There were three wells for each treatment in a given experiment for each biological replicate; at least three biological replicates were performed for each treatment.

The total number of EdU-positive cells was divided by the total number of nuclei in an image to yield the percentage of cells in S-phase to determine cell cycle exit. To determine the number of MyHC-positive cells, images from the red and blue channels were superimposed. A nucleus was considered to be MyHC-positive if it was contained within a cell emitting red fluorescence above background levels. To monitor cell fusion and calculate the differentiation index, the phase-contrast image was superimposed with the DAPI and MyHC channels. Nuclei were considered to be in fused cells if the nuclei were in a MyHC-positive cell containing three or more nuclei. MyHC-positive cells containing two or fewer nuclei were not considered to be myotubes. The number of nuclei in fused cells was divided by the total number of nuclei to yield the percentage of nuclei contained within fused cells.

### Western blots

H2K cells were differentiated in six-well dishes with the appropriate pharmacological agent and lysates were harvested at 0, 12, 24 and 36 h after differentiation. Lysates from 50,000 cell equivalents were separated by SDS-PAGE, transferred to a nitrocellulose membrane and blocked for 2 h at room temperature or overnight at 4°C in 3% BSA in PBST (PBS containing 0.1% Tween 20). Primary antibodies used were rabbit antibodies against ERK (1:1000; Cell Signaling Technologies, 9102), phospho-ERK (1:1000; Cell Signaling Technologies, 4377), p38 (1:500; Cell Signaling Technologies, 9212), phospho-p38 (1:500; Cell Signaling Technologies, 4511), H4 (1:50,000; Millipore, 05-858) and acetyl-H4K5 (1:1000; Millipore, 07-327), gamma-tubulin (1:10,000; Sigma-Aldrich, T6557). The blots were washed five times in PBST and incubated with a goat anti-rabbit HRP secondary antibody or goat anti-mouse secondary antibody (1:10,000). The blots were treated with ECL chemiluminescence detection reagent (GE healthcare, product # RPN2106V1 and RPN2106V2) and imaged using the Bio-Rad Chemidoc system (Bio-Rad Laboratories). Volume analysis was performed using ImageLab software (Bio-Rad Laboratories) as per the manufacturer's instructions.
